# Aberrant expression of CD54 detected by flow cytometry is a characteristic of B-lymphoma cells in bone marrow specimens

**DOI:** 10.1186/s12885-021-09061-3

**Published:** 2021-12-08

**Authors:** Wei Wang, Yan Li, Haval Ali, Linjun Zhao, Di Mei, Wenqing Hu, Bin Jiang

**Affiliations:** 1 Department of Pathology and Laboratory Medicine, The University of Texas Health Science Center at Houston, 6431 Fannin Street, Houston, TX USA; 2 Department of Hematology, Peking University International Hospital, Zhong-Guan-Cun Life Science Park Road, Beijing, China; 3 Department of Lymphoma, Peking University International Hospital, Zhong-Guan-Cun Life Science Park Road, Beijing, China

**Keywords:** CD54, Flow cytometry, B-cell non-Hodgkin lymphoma, Bone marrow involvement

## Abstract

**Background:**

Flow cytometry (FC) is a popular method to detect bone marrow (BM) involvement in patients with B-cell non-Hodgkin lymphoma (B-NHL). The majority of screen panels of FC still rely on finding monoclonal B-cells, e.g., B-cells with immunoglobin (Ig) light-chain restriction, which has many limitations. Therefore, exploring new markers is warranted.

**Methods:**

A total of 52 cases of B-NHL with BM involvement were collected. The median age was 60 years. Out of these 52 cases, 34 were male, and 18 were female. A 10-color FC panel was used to detect the expression of CD54 on lymphoma cells. The expression of CD54 was calculated as the mean fluorescence index ratio (MFIR) and was described as the mean ± standard error of the mean (SEM).

**Results:**

Up to 18/52 (34.62%) of BM specimens abnormally expressed an increased level of CD54, including 1/10 cases of chronic lymphocytic leukemia/small lymphocytic lymphoma (CLL/SLL), 9/13 cases of mantle cell lymphoma (MCL), 2/14 cases of follicular lymphoma (FL), 5/9 cases of marginal zone lymphoma (MZL), and 1/3 cases of high-grade B-NHL (HG B-NHL). The expression level of CD54 was significantly increased in MCL cases (53.41 ± 11.04) compared with CLL/SLL cases (11.66 ± 2.79) and FL cases (13.49 ± 2.81). The lowest percentage of CD54-positive B-cells attained 0.13%. In 5/9 cases of MZL and 1/3 cases of HG B-NHL, increased expression of CD54 was the only abnormal immunophenotype detected besides Ig light-chain restriction. No aberrant CD54 expression was identified by FC in lymphoplasmacytic lymphoma (LPL) (0/2) and Burkitt lymphoma (BL) (0/1) cases. Aberrant expression of CD54 was not related to plasma cell differentiation.

**Conclusion:**

Lymphoma cells, especially in MCL and MZL cases, frequently show increased expression of CD54. Such aberrant expression is not related to plasma cell differentiation. We highly recommend adding CD54 to the FC screening panel to detect BM involvement in patients with B-NHL.

## Background

Involvement of bone marrow (BM) by B-cell non-Hodgkin lymphoma (B-NHL) is correlated with poor outcomes [1–5]. At present, flow cytometry (FC) is a rapid, sensitive, reliable, and widely used method to determine the extension of B-NHL to the BM [6–12]. Detecting BM involvement by FC is considered an independent predictor of worse outcomes in specific subtypes of B-NHL (such as diffuse large B-cell lymphoma (DLBCL)) [8, 11].

In FC, the hallmark of B-NHL diagnosis is the presence of monoclonal B-cells, which can be detected as immunoglobulin (Ig) light-chain restriction. Hence, numerous FC screening panels designed to detect B-NHL include Ig light-chain antibodies. Ig light-chain restriction, however, is a nonspecific phenomenon; it also presents in reactive/benign B-cell proliferation [13–18]. To further complicate matters, some subtypes of B-NHL such as DLBCL and high-grade B-NHL (HG B-NHL) lack surface light-chain, and they do not show surface light-chain restriction in FC [19, 20]. On that account, exploring novel markers to distinguish between reactive/benign mature B-cells and lymphomatous B-cells is crucial.

CD54, Intracellular adhesion molecule-1 (ICAM-1), is expressed on leukocytes [21]. It functions as an adhesive and co-stimulatory molecule [22]. It has an essential role in lymphocyte adhesion, homing, activation, and tumor immune response [23, 24]. In a previous study, our group observed that 52.17% of DLBCL cases with BM involvement expressed an increased level of CD54, in contrast to normal mature B-cells, which expressed a low level of CD54 [25]. In this study, we measured the level of CD54 expression on the lymphoma cells in BM specimens involved by other subtypes of B-NHL.

## Methods

### Patients

The Hospital Ethical Committee approved the study based on the guidelines of the Helsinki Declaration of 2008. Fifty-two BM specimens from B-NHL patients with BM involvement were collected at Peking University International Hospital from 2019 to 2021. The diagnostic criteria of B-NHL were based on the latest WHO Classification of Tumours of Haematopoietic and Lymphoid Tissues recommendations [26, 27]. Thirty-six cases were newly diagnosed, and 16 cases were previously treated with residual disease in their bone marrow specimens. Out of 52 cases, 34 were males, and 18 were females with a median age of 60 years (28–81 years). Ten cases were chronic lymphocytic leukemia/small lymphocytic lymphoma (CLL/SLL), 13 cases were mantle cell lymphoma (MCL), 14 cases were follicular lymphoma (FL), 9 cases were marginal zone lymphoma (MZL), 2 cases were lymphoplasmacytic lymphoma (LPL), 3 cases were HG B-NHL, and 1 case was Burkitt lymphoma (BL) (Table 1). The clinical and epidemiological information was retrieved from the electronic medical records.

**Table 1:** Clinical and epidemiological information of patients with bone marrow involvement

	Median age (range)	Gender (male/female)	Cases with BM involvement	Cases with high CD54
CLL/SLL	67 (58–76)	8/2	10	1
MCL	65 (43–76)	11/2	13	9
FL	49 (29–69)	3/11	14	2
MZL	70 (44–74)	6/3	9	5
LPL	(65–81)	2/0	2	0
HG B-NHL	46 (38–80)	3/0	3	1
BL	28	1/0	1	0

*BM* bone marrow, *CLL/SLL* Chronic lymphocytic leukemia/small lymphocytic lymphoma, *MCL* Mantle cell lymphoma, *MZL* Marginal zone lymphoma, *FL* Follicular lymphoma, *LPL* Lymphoplasmacytic lymphoma, *HG B-NHL* High-grade B-cell Non-Hodgkin lymphoma, *BL* Burkitt lymphoma

In the meanwhile, 35 B-NHL patients without BM involvement were observed in parallel. Out of 35 cases, 17 were males, and 18 were females with a median age of 59 years (32–84 years). Eight cases were MCL, 15 cases were FL, 11 cases were MZL, and 1 case was HG B-NHL.

### Flow cytometry analysis

BM specimens (5 × 10^5^ white blood cells per heparin anticoagulant tube) were collected and stained no later than 8 h after the procurement. As described in a previous study, similar antibody panels and a 4-laser, 10-color Becton Dickinson (BD) FACSCanto™ flow cytometer (San Diego, California, USA) were used [25]. The tube with CD54 was designed as CD54 BV421/CD34 BV510/CD10 BV405/Bcl-2 FITC/CD38 PE/CD5 PerCP-Cy5.5/CD20 PE-Cy7/CD138 APC/CD19 APC-R700/CD45 APC-H7. Similar gating strategies as described previously were used [25]. Forward scatter (FSC)/side scatter (SSC) was used to exclude debris and dead cells, FSC height/ FSC area to identify single cells, then CD45/CD19/CD20 to gate on lymphoma cells. If this strategy failed to highlight lymphoma cells, other specific markers were utilized, e.g., CD5/CD19 to gate on lymphoma cells in CLL/SLL and MCL cases and CD10/CD19 in FL cases. The normal ratio of kappa/lambda on mature B-cells of BM specimens is 0.5–3.0:1 as described previously [28].

The expression of CD54 was calculated as the mean fluorescence index ratio (MFIR) (MFI of CD54 BV421/negative control BV421) as described previously [25].

### Bone marrow aspirate and biopsy

BM trephine biopsy, aspirate smears, and clot sections were also collected. BM aspirate smears were stained with Wright-Giemsa. BM biopsy specimens were fixed, paraffin-embedded, and then stained with Hematoxylin & eosin (H&E). Immunohistochemical (IHC) stain was performed on BM biopsy specimens using CD3, CD5, CD10, CD20, PAX-5, MUM-1, and CD138.

### Diagnostic criteria of BM involvement

Two hematopathologists reviewed the BM biopsy specimens. If any discordance was encountered, a third hematopathologist was consulted. As reported previously, the same diagnostic criteria to identify BM involvement in patients with B-NHL were used [29].

### Statistics

The CD54 MFIR of lymphoma cells in different patient groups were presented as the mean ± standard error of the mean (SEM). The formula used to calculate the percentage of CD54 expression was: (the number of lymphoma cells with increased expression of CD54/the number of CD45 positive cells) × 100%. A one-way analysis of variance (ANOVA) was used to evaluate the difference of CD54 MFIR between four subgroups, e.g., CLL/SLL, MCL, FL, and MZL. While LPL, HG B-NHL, and BL were not included due to low case numbers. A *p*-value lower than 0.05 was considered statistically significant.

In this study, 27.45 was used as a cut-off value of CD54 MFIR. This value was calculated in the previous study by our group using 10 BM specimens from patients without lymphoma. The statistical method was the Receiver Operating Characteristic (ROC) Curve [25].

All the data were analyzed using GraphPad Prism 6 (GraphPad Software, San Diego, CA, USA).

## Results

### The BM results of B-NHL cases with BM involvement

In 39/52 B-NHL cases, BM involvement was confirmed by BM biopsies. In the rest of the cases, the BM biopsies didn’t show evidence of lymphoma; however, the BM involvement was confirmed by detecting Ig light-chain restriction and Ig heavy chain rearrangement using FC and PCR, respectively.

In the BM specimens of the 52 B-NHL cases, the median lymphocytes number collected by flow cytometer was 23,061 (1127 – 318,940), which was considered adequate for FC analysis. Lymphoma cells of the 52 cases were positive for the light-chain restriction in FC analysis. As mentioned earlier, the BM biopsies of 13 B-NHL cases did not show evidence of BM involvement. Table 2 lists the immunophenotype of these 13 B-NHL cases. Among them, 12 cases showed surface Ig light-chain restriction, while 1 case showed cytoplasmic Ig light-chain restriction, which was HG B-NHL. The lymphoma cells of the 5 MCL cases and 1 HG B-NHL case abnormally expressed CD5, while the 2 FL cases expressed CD10.

**Table 2:** Immunophenotype of thirteen patients diagnosed as BM involvement by using flow cytometry

Number	Lymphoma cells/nucleated cells in BM (%)	Subtype	Light-chain restriction	Aberrant antigen expression	CD54 MFIR
1	0.13	MCL	+	CD5	47.69
2	1.96	MCL	+	CD5	17.11
3	3.71	MCL	+	CD5	91.96
4	0.40	MCL	+	CD5	87.28
5	1.38	MCL	+	CD5	9.23
6	2.23	FL	+	CD10	11.30
7	1.25	FL	+	CD10	2.13
8	0.18	MZL	+	none	99.71
9	2	MZL	+	none	52.63
10	1.30	MZL	+	none	43.19
11	0.36	MZL	+	none	4.53
12	0.40	HG B-NHL	+	none	5.39
13	0.50	HG B-NHL	+^a^	CD5	44.72

*BM* bone marrow, *MFIR* mean fluorescence index ratio, *CLL/SLL* Chronic lymphocytic leukemia/small lymphocytic lymphoma, *MCL* Mantle cell lymphoma, *MZL* Marginal zone lymphoma, *FL* Follicular lymphoma, *LPL* Lymphoplasmacytic lymphoma, *HG B-NHL* High-grade B-cell Non-Hodgkin lymphoma, *BL* Burkitt lymphoma

^a^This HG B-NHL lacked surface light-chain restriction, while had cytoplasmic light-chain restriction

### The expression of CD54 in different subtypes of B-NHL in BM involvement specimens

Clinical information of different subgroups is presented in Table 1. There was a total of 18 cases aberrantly expressing CD54, which included 10% (1/10) of CLL/SLL cases, 69.23% (9/13) of MCL, 14.29% (2/14) of FL, 55.56% (5/9) of MZL, and 33.33% (1/3) of HG B-NHL. Seven out of the 13 cases diagnosed with BM involvement by FC revealed increased CD54 expression (Table 2). FC identified no aberrant CD54 expression in the cases of LPL (0/2) and BL (0/1). Out of these 18 cases, 12 were prior to treatment, and 6 were after treatment. All cases expressing an increased level of CD54 are presented in Table 3.

**Table 3:** The characteristic of eighteen cases abnormally expressing CD54

Number	Subtype	Treatment status Before/After	Lymphoma cells expressing increased level of CD54/nucleated cells in BM (%)	CD54 MFIR
1	CLL/SLL	Before	12.32	29.30
2	MCL	After	25.25	108.12
3	MCL	Before	0.13	47.69
4	MCL	Before	21.79	31.70
5	MCL	After	3.71	91.96
6	MCL	Before	21.28	131.19
7	MCL	Before	9.34	66.23
8	MCL	Before	4	38.21
9	MCL	Before	76.50	32.81
10	MCL	After	0.40	87.28
11	FL	After	9.78	35.06
12	FL	After	3.80	28.01
13	MZL	Before	0.18	99.71
14	MZL	Before	6.17	30.69
15	MZL	Before	2	52.63
16	MZL	Before	13.80	52.77
17	MZL	After	1.30	43.19
18	HG-B-NHL	Before	0.50	44.72

*BM* bone marrow, *MFIR* mean fluorescence index ratio, *CLL/SLL* Chronic lymphocytic leukemia/small lymphocytic lymphoma, *MCL* Mantle cell lymphoma, *MZL* Marginal zone lymphoma, *FL* Follicular lymphoma, *HG B-NHL* High-grade B-cell Non-Hodgkin lymphoma

**Fig. 1:**
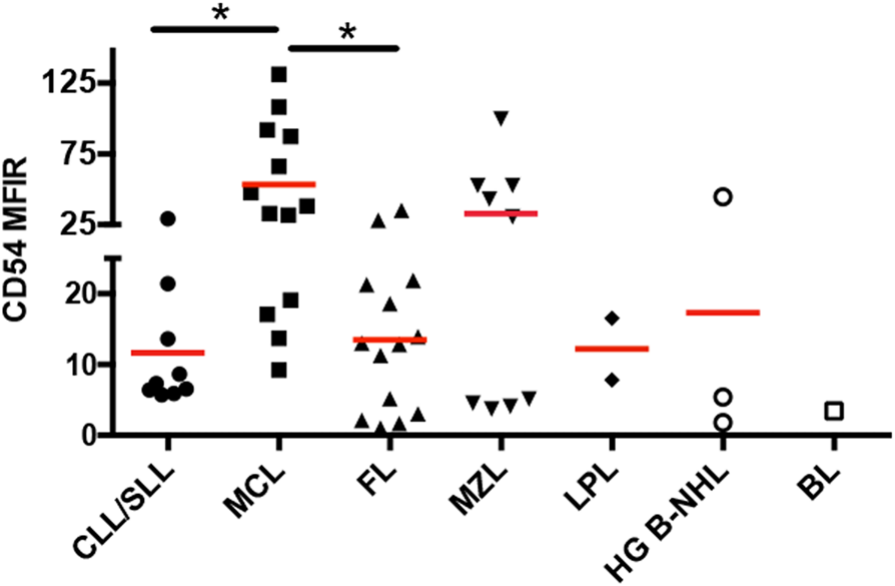
Expression levels of CD54 in different subtypes of B-NHL. The results of flow cytometry show CD54 expression in different subtypes of B-NHL with BM involvement. MCL cases significantly expressed a higher level of CD54 in comparison to CLL/SLL and FL. ANOVA was used. Horizontal bars present the mean values

The CD54 MFIR of CLL/SLL was 11.66 ± 2.79, MCL was 53.41 ± 11.04, FL was 13.49 ± 2.81, MZL was 32.94 ± 10.94, HG B-NHL was 17.31 ± 13.75, and BL was 3.44. CD54 expression was significantly higher in MCL compared with either CLL/SLL or FL (*p* < 0.05) (Fig. 1).

### The expression of CD54 in patients without BM involvement

Expression of CD54 was observed in 35 BM specimens from B-NHL patients without BM involvement; however, none of them aberrantly expressed CD54 (10.77 ± 1.31).

### The expression patterns of CD54 on lymphoma cells

**Fig. 2:**
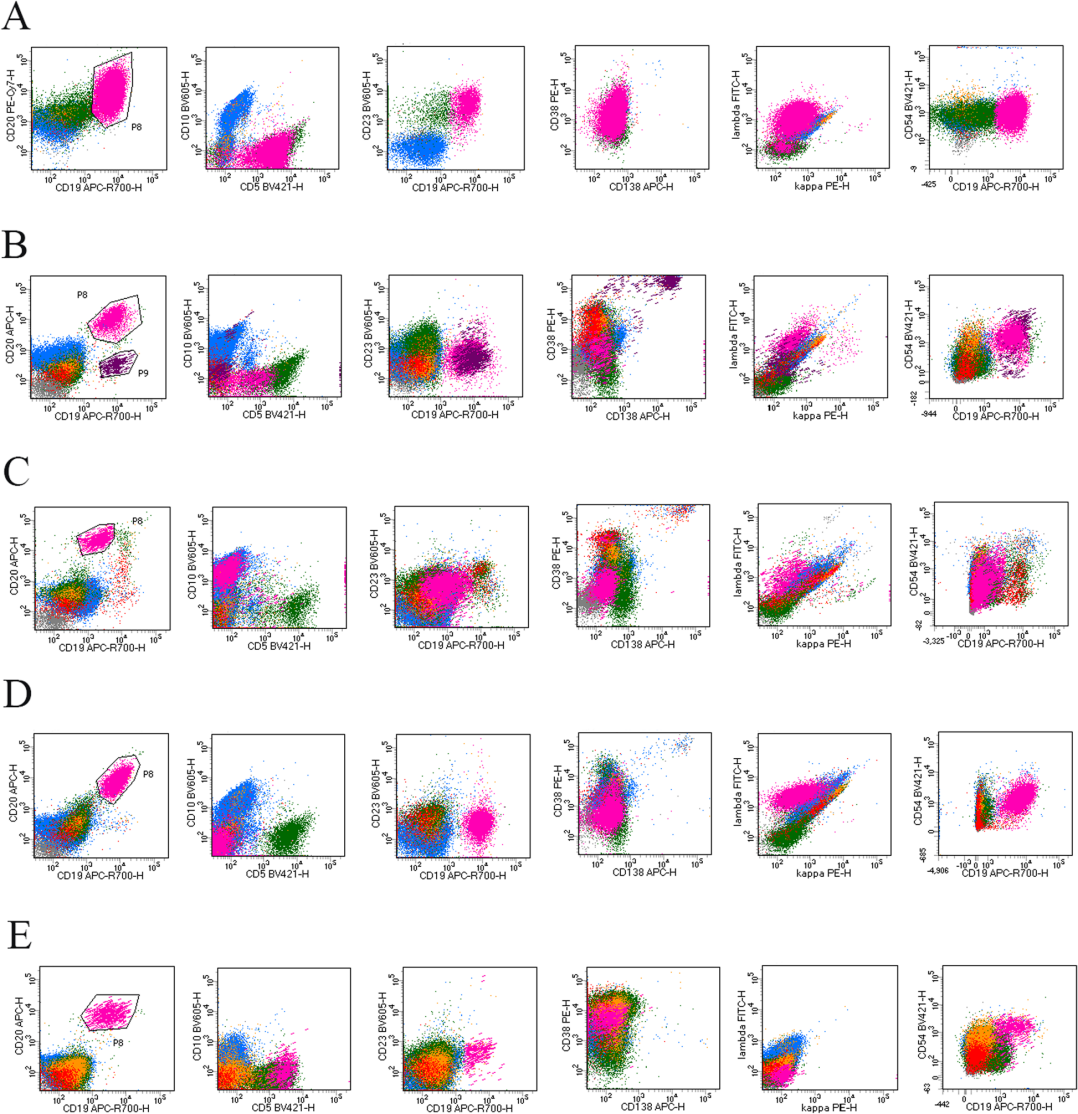
Expression profile of CD54 in different subtypes of B-NHL. The results of flow cytometry for B-NHL patients with BM involvement are present. In each figure, lymphoma cells are shown in pink color, other mature lymphocytes in green color, normal plasma cells in purplish-red color, granulocytes in blue color, monocytes in earthly yellow color, cells in the blast gate and hematogone gate in red color, and other CD45 negative cells in gray color. **A**: from a patient with CLL/SLL. Lymphoma cells have light-chain restriction and are positive for CD45, CD19, CD20, CD5, CD23, and CD54, negative for CD38 and CD138. **B**: from a patient with MCL. Lymphoma cells have light-chain restriction, and are positive for CD45, CD19, CD20, and CD54, dim for CD5, negative for CD5, CD23, CD38, and CD138. **C**: from a patient with FL. Lymphoma cells have light-chain restriction, and positive for CD45, CD20, CD10, and CD54 (partial), dim to negative for CD19, negative for CD5, CD23, CD38, and CD138. **D**: from a patient with MZL. Lymphoma cells have light-chain restriction, and are positive for CD45, CD19, CD20, and CD54, negative for CD5, CD10, CD38, and CD138. **E**: from a patient with HG B-NHL. Lymphoma cells are absent of surface light-chain; positive for CD45, CD19, CD20, CD5, and CD54, negative for CD10, CD38, and CD138

Abnormal expression patterns of CD54 on lymphoma cells in different subtypes of B-NHL are shown in Fig. 2. Figure 2A shows a smear pattern in which the expression of CD54 is extended uninterruptedly from the normal level to the abnormal level. Figure 2C shows a separated pattern in which the population with increased CD54 expression is separated from the population with normal CD54 expression. Figure 2B, D, and E show a uniform pattern, in which there is only one population of lymphoma cells with increased CD54 expression.

### The lowest percentage of CD54-positive B-cells in BM involvement cases

The mean percentage of CD54-positive B-cells was 11.79% (0.13–76.50%) (Table 3). The lowest percentage of CD54-positive B-cells was 0.13%, and it was in an MCL case.

### The correlation between the increased expression of CD54 and plasma cell differentiation

Since normal plasma cells highly express CD54 [25], the correlation between the aberrant expression of CD54 and plasma cell differentiation of lymphoma cells was investigated. The diagnostic criteria described by Jourdan M et al. to detect plasma cell differentiation based on morphology and immunophenotype were used [30]. Firstly, there was no morphologic evidence supporting plasma cell differentiation in all 18 cases aberrantly expressing CD54. Secondly, there was no immunophenotypic evidence about plasma cell differentiation in those cases either. In CLL/SLL, MCL, MZL, FL, and HG B-NHL cases, the lymphoma cells were positive for CD20 and PAX5 and negative for CD38 and CD138 by IHC. By FC analysis, the lymphoma cells were positive for CD20, negative for CD138, and few of them were dim for CD38. Two FL cases were dim for CD19 by FC, which is frequently seen in FL. Subsequently, it was concluded that the increased expression of CD54 on lymphoma cells is not related to plasma cell differentiation.

## Discussion

In contrast to BM biopsy, FC is more sensitive in detecting lymphoma cells, especially when the tumor burden is low [31, 32]. FC is also cheaper and faster than molecular analysis. At present, FC is widely used as a complementary method to determine BM involvement in patients with B-NHL.

From a practical standpoint, an initial screening panel should be sensitive enough to detect lymphoma cells. Although numerous B-NHL FC panels have been published, most of them used five or fewer colors. The following studies have used multiple colors in their screening tubes to detect B-NHL: the Euroflow panel (kappa, lambda, CD19, CD20, CD45, CD5, and CD38) [33]; the Toronto/Lund panel (BM specimen: kappa, lambda, CD19, CD20, CD45, CD5, CD10, and CD34, blood/tissue/body fluids specimen: kappa, lambda, CD19, CD20, CD45, CD5, CD10, CD38, CD23) [34, 35]; and the London Health Science Center/Toronto panel (kappa, lambda, CD19, CD20, CD45, CD5, CD10, live/dead dye) [36]. These panels rely on light-chain restriction to detect monoclonal B-cells and confirm BM involvement. It is well-known that Ig light-chain restriction also presents in reactive/benign B-cell proliferation [13–18]. Some subtypes of B-NHL lack the surface expression of light-chain, so they do not show surface light-chain restriction in FC analysis [19, 20]. Thus, utilizing light-chain restriction as a sole criterion to identify B-cell lymphomas has many limitations. Detection of abnormal immunophenotype is another tool to highlight lymphoma cells. Detecting lymphoma cells using FC is straightforward for certain subtypes of B-NHL due to their abnormal immunophenotypes. For example, CLL/SLL cells are positive for CD5, CD23, CD200, dim for CD20 and CD81, and negative for FMC-7 [37]; in MCL, lymphoma cells are positive for CD5, and negative for CD23 [38, 39]; and in FL, lymphoma cells are positive for CD10 and CD20, and negative for CD34 and TdT [33, 40]. While in other B-NHL subtypes, such as MZL and LPL, the light-chain restriction is the only abnormality detected by FC. Immunophenotypic abnormalities of different B-NHL subtypes are overly heterogeneous; hence, including all markers in one screening tube with kappa and lambda is difficult. Therefore, the need to explore a new marker that can detect different subtypes of lymphoma cells is justified.

As previously reported, the lymphoma cells in BM specimens from patients with DLBCL frequently express an increased level of CD54 (52.17%), especially in the non- germinal center B-cell (non-GCB) subtype (72.73%) [25]. In this study, we found that in BM involvement cases, 62.93% of MCL, 55.56% of MZL, 33.33% HG B-NHL, 14.29% of FL, and 10% of CLL/SLL aberrantly expressed CD54. Furthermore, MCL cases significantly expressed a higher level of CD54 in comparison to CLL/SLL and FL cases. In LPL cases, lymphoma cells expressed a low level of CD54. However, the number of LPL cases in this study is too low to conclude. In BL, lymphoma cells expressed a low level of CD54, which is consistent with what Schniederjan SD et al. previously reported [41]. We observed that B-cell lymphoblastic leukemia (B-ALL) expressed a low level of CD54 (data is not shown here in detail). This observation leads us to assume that B-cell lymphoblastic lymphoma (LBL) should also express a low level of CD54. Based on the above findings, CD54 can be used to gate on lymphoma cells, especially in MCL and MZL cases. Then, the Ig light-chain restriction can be detected to confirm the presence of lymphoma cells in BM specimens.

In some subtypes of B-NHL without obvious abnormal immunophenotype, the detection sensitivity of FC relying on light-chain restriction is as low as 0.1% [33]. In this study, the lowest percentage of CD54-positive B-cells reached 0.13%; this supports the notion that detecting lymphoma cells by using CD54 is as reliable as detecting Ig light-chain restriction. However, more cases need to be enrolled to confirm this assumption. Another promising finding is that one case of HG B-NHL abnormally expressed CD54. It is well-known that lymphoma cells of HG B-NHL frequently lack surface light-chain expression. The expression of the cytoplasmic light chain can only be detected after permeabilizing these cells. This procedure is time-consuming and decreases the detection sensitivity because lymphoma cells of HG B-NHL are usually large and fragile, and the permeabilization process destroys many lymphoma cells. Thus, CD54 could be used to identify lymphoma cells in HG B-NHL cases, and the diagnosis of BM involvement might be made even without testing light-chain restriction. To confirm this postulation, more HG B-NHL cases need to be included.

In most cases, it is easy to interpret the increased expression of CD54. However, more caution should be exercised in particular circumstances, where the increased level of CD54 is only present in a subpopulation of lymphoma cells. Furthermore, it is challenging to distinguish the abnormal expression of CD54 in rare cases, especially when the CD54 is expressed as a smear pattern. To interpret the expression of CD54 on lymphoma cells precisely, normal immature and mature B-cells can be used as the internal negative controls and normal plasma cells as the internal positive control.

As explained above, the increased expression of CD54 on lymphoma cells is not related to plasma cell differentiation. Cells with plasma cell differentiation have plasmacytoid morphology, highly positive for CD38, positive for CD138 and MUM-1, and negative for PAX5 and CD20. Although some B-NHL cases were dim for CD38, it is not difficult to interpret plasma cell differentiation in B-NHL cases. However, there are overlaps of the immunophenotype of preplasmablasts (CD20 dim/−, CD38-, CD138-), plasmablasts (CD20-, CD38+, CD138-) [30] and CLL/SLL. Therefore, in CLL/SLL cases, the morphology and immunophenotype (CD20 +/dim, CD138-, MUM-1- detected by FC or IHC) are used to rule out plasma cells differentiation. For this reason, we suggest adding CD38 and CD138 with CD54, CD19, and CD20 in the same tube to differentiate the abnormal cells from normal plasma cells.

Since CD54 involves lymphocytic homing and activation, we assume that increased expression of CD54 might predict chemotherapy resistance. In this study, 6/18 cases with increased expression of CD54 were after treatment. However, a large cohort study is needed to confirm this hypothesis.

More cases, especially MZL, LPL, and HG B-NHL cases, need to be enrolled in future studies. Currently, a study detecting CD54 expression in lymphoid tissues is being conducted to obtain more clarification about CD54 expression on lymphoma cells.

## Conclusions

In BM specimens, increased expression of CD54 on mature B-cells is abnormal. Lymphoma cells, especially in MCL and MZL cases, frequently show increased expression of CD54. Such increased expression is not related to plasma cell differentiation. CD54 can be added to the FC screening panels used to detect BM involved by B-NHL.

## Data Availability

All data generated or analyzed during this study are included in this published article.

## Abbreviations

BM: Bone marrow
NHL: Non-Hodgkin lymphoma
FC: Flow cytometry
DLBCL: Diffuse large B-cell lymphoma
Ig: Immunoglobin
ICAM-1: Intracellular adhesion molecule-1
CLL/SLL: Chronic lymphocytic leukemia/small lymphocytic lymphoma
MCL: Mantle cell lymphoma
MZL: Marginal zone lymphoma
FL: Follicular lymphoma
LPL: Lymphoplasmacytic lymphoma
HG B-NHL: High-grade B-cell Non-Hodgkin lymphoma
BL: Burkitt lymphoma
MFIR: Mean fluorescence intensity ratio
SEM: Standard error of the mean
IHC: Immunohistochemical
ANOVA: A one-way analysis of variance
ROC: Receiver Operating Characteristic
non-GCB: Non-germinal center B-cell
B-ALL: B-cell lymphoblastic leukemia
LBL: B-cell lymphoblastic lymphoma
